# Deep Learning-Based Diagnosis of Femoropopliteal Artery Steno-Occlusion Using Maximum Intensity Projection Images of CT Angiography

**DOI:** 10.3390/tomography11090104

**Published:** 2025-09-08

**Authors:** Wonju Hong, Jaewoong Kang, So Eui Kim, Taikyeong Jeong, Chang Jin Yoon, In Jae Lee, Lyo Min Kwon, Bum-Joo Cho

**Affiliations:** 1 Department of Radiology, Hallym University Sacred Heart Hospital, College of Medicine, Hallym University, Anyang 14068, Republic of Korea; wj.jenny29@gmail.com (W.H.);; 2 Medical Artificial Intelligence Center, Hallym University Medical Center, Anyang 14068, Republic of Korea; jaewoong@hallym.or.kr (J.K.);; 3 Department of Biomedical Informatics, Hallym University Chuncheon Sacred Heart Hospital, Chuncheon 24253, Republic of Korea; 4 School of Artificial Intelligence Convergence, Hallym University, Chuncheon 24252, Republic of Korea; 5 Department of Radiology, Seoul National University College of Medicine, Seoul National University Bundang Hospital, Seongnam 13620, Republic of Korea; 6 Department of Ophthalmology, Hallym University Sacred Heart Hospital, College of Medicine, Hallym University, Anyang 14068, Republic of Korea

**Keywords:** artificial intelligence, deep learning, computed tomography angiography, peripheral arterial disease, lower extremity

## Abstract

**Background/Objectives**: To develop and validate deep learning-based models for detecting significant steno-occlusion (SSO)—defined as luminal narrowing greater than 50%—of the femoropopliteal arteries using maximum intensity projection (MIP) images from lower extremity CT angiography (CTA). **Methods**: This retrospective study utilized MIP images of lower extremity CTA performed between January 2021 and December 2023 for internal model development. Deep learning-based models were developed sequentially to diagnose SSO: screening with single anteroposterior image, followed by four-segment rotational analysis that divided each femoropopliteal artery into four segments and incorporated multi-angle images. Given the cropped images and the shape of stenosis, models were trained to classify the presence of SSO. A temporal validation dataset comprised MIP images from lower extremity CTA performed between January and June 2024. **Results**: In total, 56,496 segment images from 642 patients (mean age: 68.2 ± 13.5 years; 472 men) were included in the internal dataset. In the single-image analysis, RDNet achieved the highest mean AUC of 0.886 for SSO detection. In the four-segment rotational analysis, RDNet also demonstrated the highest mean AUC, reaching 0.964 in both half-set and full-set approaches. While RDNet recorded the highest mean AUC, all other models showed improved AUCs as the number of input images increased (*p* < 0.05). In the temporal validation dataset, RDNet again achieved the highest mean AUC (0.959) in the half-set analysis. **Conclusions**: The deep learning-based model, particularly RDNet, demonstrated excellent performance in detecting SSO of peripheral arteries on MIP images from lower extremity CTA.

## 1. Introduction

Stenosis or occlusion of the femoropopliteal artery is a key imaging finding in peripheral arterial disease (PAD) and acute limb ischemia (ALI) of the lower extremities. PAD, primarily caused by atherosclerosis, varies in severity from asymptomatic narrowing to severe tissue damage or necrosis due to chronic ischemia [1]. As the global population ages, PAD prevalence is increasing, currently affecting approximately 236 million individuals worldwide [2,3]. ALI, a medical emergency, is characterized by a sudden reduction in blood flow to the lower extremities, requiring rapid diagnosis and treatment [4].

Lower extremity CT angiography (CTA) is widely employed as a noninvasive, high-resolution diagnostic tool for peripheral arterial disease. It enables accurate assessment of stenosis and occlusion, particularly valuable in emergency scenarios [5,6]. However, interpreting lower extremity CTA requires thorough evaluation of the entire peripheral arterial system, both in non-contrast phases for calcifications and arterial phases for assessing luminal patency, becoming particularly challenging with extensive or multifocal lesions. To aid interpretation, three-dimensional maximum intensity projection (MIP) images generated from raw data automatically remove bones and display calcifications as dense lesions, facilitating the detection of severe stenosis or diffuse lesions [7]. Nonetheless, increasing imaging volumes, a shortage of specialized cardiovascular radiologists, and rising clinical workloads complicate interpretation [8].

Recently, various artificial intelligence (AI)-assisted diagnostic systems have been developed and implemented in clinical practice, demonstrating high performance in radiology. However, research focusing specifically on peripheral artery stenosis using CTA remains limited [9]. Previous studies have developed AI models using CTA axial or magnetic resonance (MR) images [10,11], but very few studies have explored the use of MIP images [12,13]. One prior study developed a vision transformer model on MIP images and reported an accuracy of 71% using a small number of images; in contrast, our work utilized a dataset with more than 400-fold greater number of images [13]. Utilizing MIP images may offer advantages such as eliminating the need for bone removal, providing clearer vessel visualization, and enabling more effective detection and segmentation of vessels with fewer image slices [14]. If deep learning-based AI could assist radiologists by pre-selecting the presence and location of significant stenotic lesions on MIP images from lower extremity CTA, it could reduce radiologists’ workloads, shorten interpretation times, and ultimately contribute to the early detection and improved treatment outcomes for PAD and ALI.

Therefore, this study aimed to develop a deep learning-based model capable of detecting significant steno-occlusion (SSO, defined as luminal narrowing greater than 50%) of femoropopliteal arteries on MIP images derived from lower extremity CTA, thus assisting radiologists in achieving rapid and accurate diagnoses.

## 2. Materials and Methods

### 2.1. Subjects

**Figure 1:**
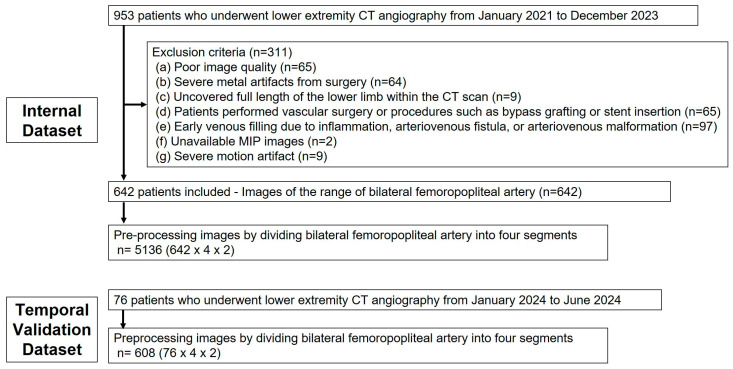
Flowchart of the construction of the dataset containing multi-angle MIP images.

This retrospective observational study was approved by the institutional review board (No. 2024-07-002). Given its retrospective design, the requirement for written informed consent was waived. The internal dataset of this study included 953 out of 1122 patients who underwent lower extremity CTA at Hallym University Sacred Heart Hospital between January 2021 and December 2023, specifically selecting those who had CTA with maximum intensity projection (MIP) images. Exclusion criteria were applied as follows: poor image quality due to inadequate contrast agent injection resulting from compromised cardiac function or outdated imaging equipment; severe metal artifacts from prior surgeries impairing vessel assessment; incomplete imaging of the full length of the lower limb; scans performed after vascular interventions such as bypass grafting or stent insertion; and cases with early venous filling due to inflammation, arteriovenous fistulas, or arteriovenous malformations (Figure 1). To validate the proposed methodology, a temporal validation dataset was compiled using the same selection criteria, consisting of 76 patients examined from January to June 2024.

### 2.2. Dataset Construction

An MIP database was constructed by prioritizing images stored within the picture archiving and communication system, excluding those meeting the exclusion criteria. Due to the high resolution and large file sizes of MIP images, there was a significant risk of prolonged training times and reduced performance in the deep learning model. To mitigate this, a specifically annotated MIP database was created for training, concentrating on regions of interest (ROIs) where peripheral arterial stenosis detection was essential. These ROIs were labeled based on the presence or absence of stenosis. The ROI was delineated using two anatomical landmarks: the femoral bifurcation and the distal end of the popliteal artery, on both the right and left sides.

The degree of stenosis and calcium classification were annotated by two radiologists (K.L.M. and H.W.) with 8 and 6 years of vascular imaging experience, respectively. In the case of disagreement, consensus was achieved through joint review and discussion. Their assessments were based on axial and coronal images from non-contrast, arterial, and delayed phases of CT angiography, supplemented by MIP images and, where applicable, conventional angiographic images obtained after intervention. Conventional angiography within 1 month was performed in 230 of the 642 patients (35.82%).

### 2.3. Image Pre-Processing

**Figure 2:**
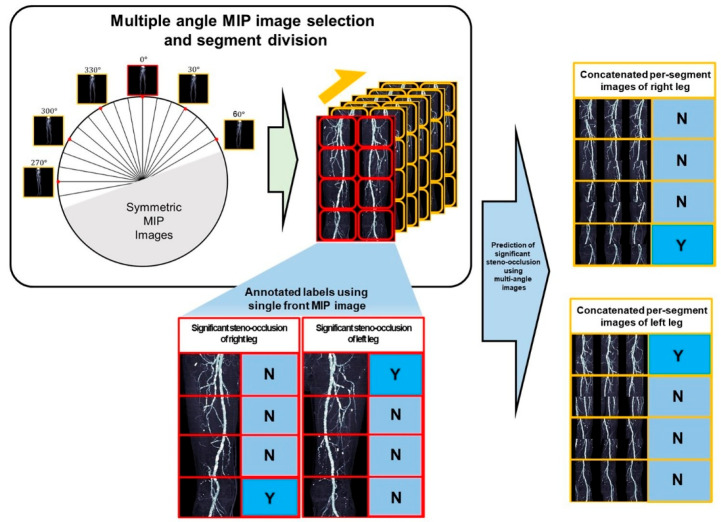
Framework for diagnosing significant steno-occlusion (SSO) using segments of multi-angle MIP images. (**Left**) From 36 omnidirectional rotational MIP images in axial view, images were selected after excluding symmetrical views and overlapping regions at the extreme ends. (**Right**) Based on the presence of SSO and segment annotations from the frontal MIP image, corresponding regions were cropped from the rotational MIP images. The resulting concatenated per-segment images were then used to infer the presence of SSO. (N: absence of SSO, Y: presence of SSO).

To facilitate stenosis detection by ROI cropping (i.e., eliminating unnecessary background from MIP images), the following pre-processing steps were conducted (Figure 2). Four anatomical reference points identifying vascular coordinates from the frontal image were used to generate ROIs in rotated MIP images. Among the 36 images acquired at 10° intervals over a full 360° rotation, each image and its counterpart rotated by 180° were mirror images; thus, only one from each pair was utilized. Furthermore, images obtained from lateral patient positions were excluded due to the overlapping of left and right legs, precluding accurate stenosis detection. Considering these conditions, three analytical approaches were employed: a single anteroposterior (AP) MIP image (single-image approach), six images selected to maximize angular intervals (half-set approach), and all 11 valid images (full-set approach). In the half-set and full-set methods, multiple images were concatenated into two rows without size reduction to form a single input image.

### 2.4. Model Design

Several models were sequentially developed to diagnose SSO of the femoropopliteal arteries:

I. Screening with a single AP projection MIP image:

A detection model was developed using a single AP projection MIP image to identify femoropopliteal artery stenosis ≥50%, defined as SSO, since stenosis exceeding this threshold is clinically associated with symptomatic disease.

II. Four-segment rotational comprehensive analysis—Per-segment analysis:

Each femoropopliteal artery was subdivided into four segments, totaling eight segments per patient for this analysis. Combinations of rotated images (each rotated by 10°) were analyzed to assess the presence of SSO in each segment. Also, we proposed a multi-region ensemble model to determine the presence of SSO in the femoropopliteal artery by aggregating the classification results from each vascular segment. The multi-region ensemble strategy defined a threshold as the minimum number of correctly classified segment-level classifiers required to predict the overall condition. The ensemble model performance was compared across different thresholds, and when all eight segment classifiers were correctly classified, the overall vascular condition could be identified with complete accuracy.

III. Subgroup analyses by calcium group, age and sex:

Patients were classified into three groups according to the extent of calcification in their femoropopliteal arteries: no calcium (0%), low calcium (<50%), and high calcium (≥50% calcification). The per-segment analysis results were evaluated separately within each calcium-level group. Additional subgroup analyses were performed according to age (≤59 years, 60–69 years, 70–79 years, and ≥80 years) and sex.

### 2.5. Deep Learning

For deep learning model development, three architectures were employed: DenseNet169, EfficientNet-B6, and RDNet. DenseNet169 features enhanced algorithms for feature representation and learning efficiency, making it effective in medical image classification tasks. EfficientNet-B6, with parameter numbers comparable to DenseNet169, is a more recent architecture that achieved superior classification performance on ImageNet. RDNet has demonstrated performance exceeding vision transformers, previously considered optimal in computer vision tasks.

Given the similar parameter count among these models, batch size selection was based on input image size rather than model type. Specifically, batch sizes were set to 16 for images sized 200 × 160 pixels, 8 for 400 × 320 pixels, and 4 for both 600 × 320 and 1200 × 320 pixels, reflecting GPU memory constraints. The categorical cross-entropy loss function and Adam optimizer were employed. An initial learning rate of 1 × 10^−4^ was utilized, decreasing by a factor of 0.1 every 10 epochs.

Early stopping was implemented from the 20th epoch onward, with a patience value of 10, tracking validation loss to determine the conclusion of training within a maximum of 100 epochs. If validation loss increased beyond the lowest loss previously recorded, the model was not saved. Thus, the final model selected corresponded to the epoch with the minimum recorded validation loss to prevent overfitting.

Gradient-weighted class activation mapping (Grad-CAM) was utilized to improve model interpretability, highlighting regions most significantly influencing model decision-making, especially areas relevant to stenosis detection.

All deep learning experiments were performed by partitioning the internal dataset into three subsets. Using three independent random samplings, the data were split in an 8:1:1 ratio into a training set for model development, an internal validation set for hyperparameter optimization, and an internal test set for performance evaluation. To ensure robustness, each experiment was repeated three times.

### 2.6. Statistical Analyses

All deep learning models underwent performance evaluation on three independent test datasets using three different random seeds. Predictive performance was quantified using the area under the receiver operating characteristic curve (AUC). Statistical analyses were conducted using python 3.10 software and scikit-learn 1.4.2 library. Continuous variables are presented as mean ± standard deviation. One-way ANOVA was performed to compare differences in performance across different deep learning architectures AUCs and accuracies of three different approaches were compared using ANOVA and two different approaches were compared using Mann-Whitney U test. We compared the AUCs among the three calcium groups using ANOVA, based on the highest diagnostic performance values obtained from DenseNet, EfficientNet, and RDNet deep learning models. A *p*-value < 0.05 was considered to indicate a significant difference.

## 3. Results

A total of 56,469 segment images from 642 patients (mean age: 68.2 ± 13.5 years; 473 men, 67.3%) were included as the internal dataset. Among these, 12,683 images (22.4%) showed significant steno-occlusion. The temporal validation dataset comprised 1705 segment images from 76 patients (mean age: 70.3 ± 13.3 years; 59 men, 77.6%), of which 435 images (25.5%) demonstrated SSO (Table 1).

I. Screening with a single AP projection MIP Image

**Figure 3:**
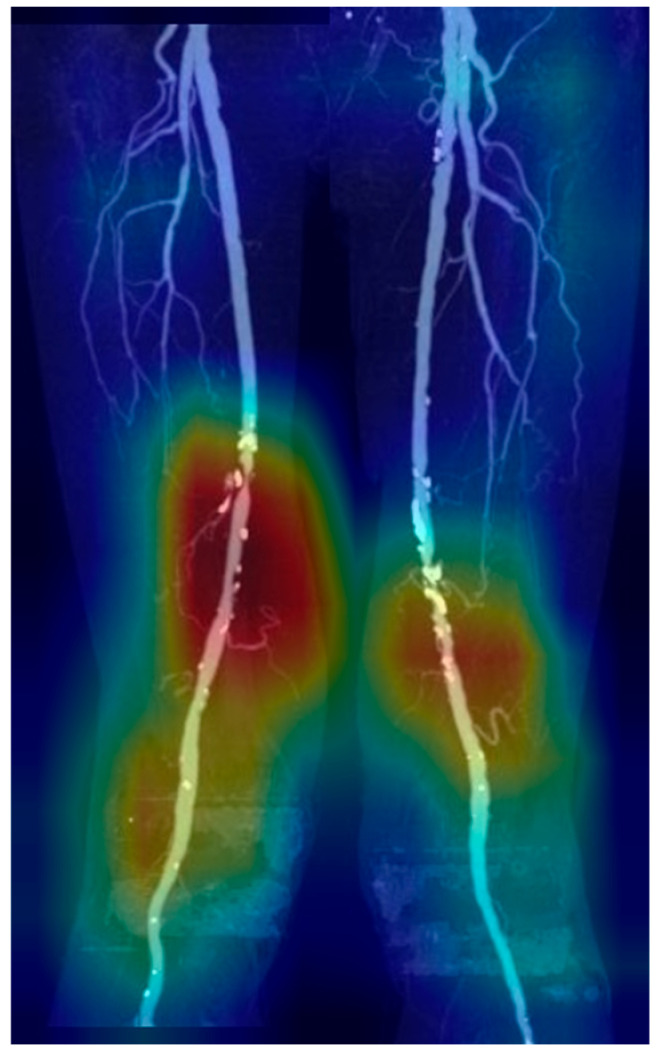
Heatmap of stenosis prediction and significant steno-occlusion (SSO) using Grad-CAM. Grad-CAM visualization of the final convolutional layer in RDNet, highlighting class-discriminative regions (red, yellow, green, blue: high to low influence in order) with SSO in femoropopliteal artery.

The predictive performance of the deep learning models on the internal test set and temporal validation dataset is presented in Table 2. In the internal test set, RDNet showed sensitivity of 0.873 ± 0.072, NPV of 0.750 ± 0.114, and AUC of 0.886 ± 0.331 for detecting SSO, surpassing those of DenseNet169 and EfficientNet-B6. The temporal validation dataset demonstrated similar results. Sensitivity of detecting SSO in RDNet was 0.925 and AUC was 0.890. Prediction of SSO was provided with a heatmap using Grad-CAM (Figure 3).

II. Four-segment rotational comprehensive analysis—Per-segment analysis

The experimental outcomes of the per-segment analysis, which predicted the presence of SSO in each segment image are summarized in Table 3 and Table 4.

In the internal test set, overall mean accuracies ranged from 85.5% to 90.2% across the three models and approaches. RDNet demonstrated the highest sensitivity in both the single-image (0.951 ± 0.052) and half-set (0.952 ± 0.022) approaches, and achieved the highest F1-score in the single-image approach (0.789 ± 0.028). EfficientNet-B6 yielded the highest accuracy in the half-set (90.2% ± 2.4%) and full-set (90.0% ± 1.1%), indicating improved performance with multiple inputs. DenseNet169 exhibited relatively consistent accuracies around 88% across approaches, with sensitivity–specificity trade-offs within a narrow range.

In the temporal validation dataset, overall accuracy was slightly lower (84.7–89.5%), but the relative performance patterns were consistent with those observed in the internal dataset. RDNet maintained the highest sensitivity (0.897), achieving comparable F1-scores to those of the other models. EfficientNet-B6 achieved the highest accuracy (89.5%) and F1-score (0.813) using the full set. DenseNet169 demonstrated accuracies between 84.7% and 89.3% across approaches, with specificity values of up to 0.903, indicating stable discriminatory ability.

AUCs of single, half-set, and full-set approaches were compared across the three models. In the internal test set, DenseNet169 and EfficientNet-B6 exhibited significantly lower predictive accuracy with the single-image approach compared to the half-set and full-set approaches, whereas this difference was not statistically significant for RDNet. In the temporal validation dataset, while RDNet recorded the highest AUC in the half-set approach, all other models demonstrated an increasing trend in AUC performance with greater numbers of utilized images

For multi-region ensemble model, lower threshold consistently yielded higher predictive performance in both the internal test set and the temporal validation dataset (Table 5). For the internal test set, the mean accuracy across all approaches was 94.3%, 89.0%, 75.3%, and 58.7% at thresholds of five through eight per-segments, respectively. In the temporal validation dataset, the corresponding accuracies were 94.0%, 87.0%, 73.5%, and 54.9%, demonstrating a consistent decline in performance as the threshold increased.

III. Subgroup analyses by calcium group, age and sex

**Figure 4:**
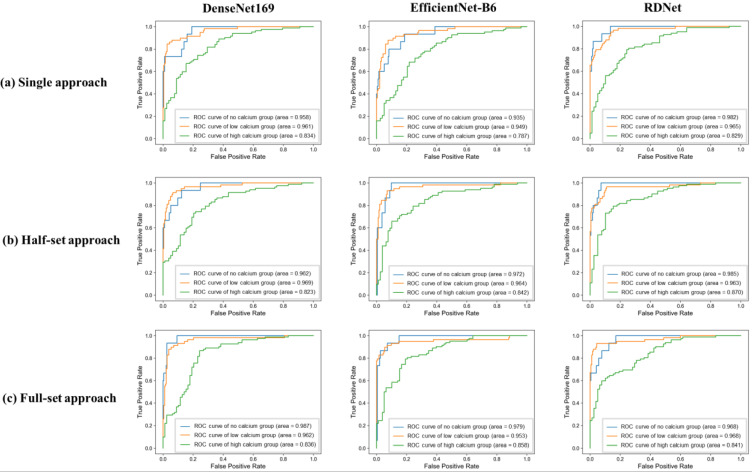
Receiver operating characteristic (ROC) curves of three deep learning models according to the degree of calcification in three approaches. In the single-image approach using only the frontal MIP image, both DenseNet169 and RDNet showed a decreasing trend in AUC with increasing calcium grade. EfficientNet-B6 showed a slight increase in AUC for the low calcium group, but the lowest AUC was observed in the high calcium group. In the half-set approach, both EfficientNet-B6 and RDNet showed a decreasing trend in AUC with increasing calcium grade, while DenseNet169 showed a slight increase in AUC for the low calcium group, but the lowest performance for the high calcium group. In the full-set approach, all deep learning models showed a consistent trend of decreasing AUC with increasing calcification.

The results for the SSO analysis stratified by calcium groups are provided in Table 6. The analysis indicated that MIP images from the high calcium group consistently demonstrated lower AUC values across all methodological approaches compared to those from low calcium and no calcium groups (*p* < 0.001). Notably, except for the DenseNet169 model in the high calcium group and the RDNet model in the low calcium group, all deep learning models recorded their lowest AUC values when using the single-image approach (Figure 4).

In the subgroup analyses of age and sex, subtle variations in values were observed, whereas no consistent trend or meaningful difference was identified. The detailed results are presented in Supplementary Tables S1 and S2.

## 4. Discussion

We developed a deep learning-based AI model to detect SSO of femoropopliteal arteries on MIP images obtained from lower extremity CTA. The models exhibited excellent performance, achieving an AUC greater than 0.8 when detecting significant steno-occlusion using a single anteroposterior projection MIP image. In the per-segment analysis, models using half-set and full-set approaches with multi-angle images performed better than those utilizing a single-image approach.

The multi-region ensemble model demonstrated that achieving concordance in five or six out of eight segment-lesions can be considered a favorable level of performance. The model maintained predictive reliability across multiple regions, although concordance across all eight segment-lesions showed relatively lower performance. The requirement for full concordance across all segment-lesions remains a limitation of the current approach. Further refinement of the ensemble methodology may improve the model’s ability to achieve more consistent and comprehensive segment-lesion concordance.

**Figure 5:**
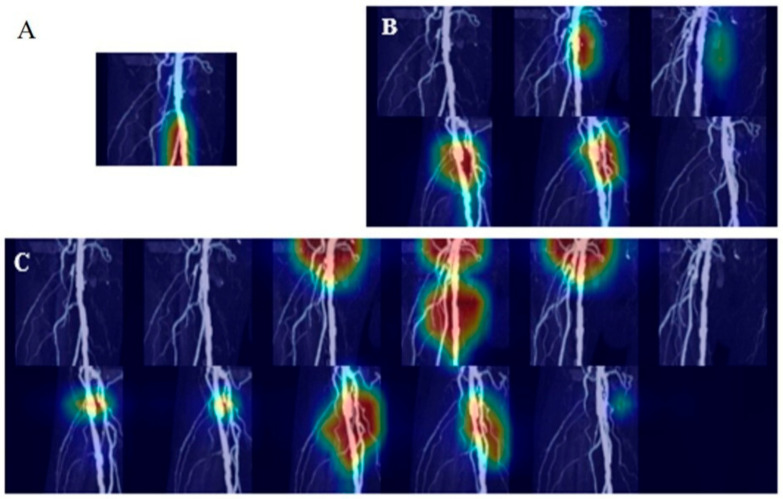
Heatmap of predicting stenosis and significant steno-occlusion (SSO) using Grad-CAM. Grad-CAM visualizations of the final convolutional layer in RDNet, highlighting class-discriminative regions (red, yellow, green, blue: high to low influence in order). (**A**) Prediction of SSO using a single image fails to localize the stenosis region, whereas (**B**,**C**), using half-set and full-set multi-angle images, respectively, successfully identifies the region by incorporating multi-angle information.

These AI models have shown considerable potential in medical imaging, especially in vascular disease detection. In our study, RDNet demonstrated superior classification accuracy in detecting SSO, outperforming DenseNet169 and EfficientNet-B6. RDNet’s strength lies in its refined dense connection mechanism, enhancing feature extraction and interpretability. To foster clinical trust, we employed Grad-CAM to visualize regions that significantly influenced the AI decision-making, as illustrated in Figure 5. Such visual explanations enable radiologists to assess the reliability of stenosis detection, underscoring the importance of transparency and clinical validation in AI adoption.

To our knowledge, this is one of the earliest attempts to predict femoropopliteal artery stenosis using AI analysis of MIP images of CTA. In 2022, Salvi et al. described their work as the first study to diagnose PAD with a vision transformer model applied to CTA MIP images, reporting 71% accuracy and 78% sensitivity based on 126 MIPs from 11 patients [13]. While their study demonstrated the feasibility of this approach in a small, exploratory setting, our work constitutes a more robust and large-scale investigation, involving over 56,000 MIP images from more than 600 patients and achieving superior diagnostic performance. PAD is relatively common, yet despite significant radiological advancements, research exploring the application of AI in lower extremity arterial assessment remains limited. Another study investigated stenosis detection using axial CTA images; however, this method may not accurately reflect disease severity, given the importance of lesion length and extent in clinical decision-making [10,15]. Nguyen et al. developed an AI model for stenosis detection using magnetic resonance (MR) angiography MIP images, but their study was limited to approximately 100 patients and did not incorporate artery segmentation [11]. However, MR angiography is less universally utilized than CTA, highlighting the necessity for further AI-focused research in CTA.

CTA tends to exaggerate calcium deposits, creating artifacts that obscure accurate assessment. The vessel lumen may appear narrower than it truly is, resulting in potential overestimation of stenosis. The presence of calcified plaques is a significant challenge in stenosis detection using CTA; however, calcium itself provides important diagnostic information regarding PAD severity. A high calcium burden is often associated with advanced age, smoking, and other cardiovascular risk factors, and the degree of calcification also serves as a determinant in decisions regarding revascularization therapy. For these reasons, calcium assessment remains a critical issue to be addressed, and we incorporated it as a baseline component in our study. Given the absence of reliable and widely available calcium reduction software (eXamine version 0.9.12.23196 (Siemens)) to serve as ground truth, calcium was retained in our analysis. Our results demonstrated that while the no- and low-calcium groups achieved AUCs exceeding 0.95, performance in the high-calcium group declined to approximately 0.83, with RDNet’s half-set approach reaching 0.870. These findings highlight the need for further methodological improvements in handling heavy calcification. Potential strategies include optimized window settings to reduce blooming artifacts on MIP images, reliable calcium reduction techniques for more accurate assessment of luminal narrowing, and additional imaging modalities such as dual-energy CT to better separate and evaluate calcified components. Moreover, AI-driven approaches on pre-processing or post-processing steps and calcium-aware training strategies could provide further performance gains [16]. Collectively, these approaches may enhance stenosis prediction in patients with significant calcium burden.

The deep learning model developed in our study can be integrated into current clinical workflows as an assistive diagnostic tool. AS MIP images are already part of standard post-processing in many radiology departments, the model requires no additional acquisition or reconstruction. By automatically identifying segments with SSO and providing pre-screening results with visual outputs such as heatmaps or segment-level predictions, the model can assist radiologists in rapidly focusing on clinically relevant areas. Furthermore, considering the high diagnostic performance of our model in identifying normal cases, an important potential application would be its integration into the Picture Archiving and Communication System. By automatically flagging patients likely to be normal or displaying sensitivity scores in the patient lists, the system could assist radiologists in prioritizing the interpretation of patients that are more likely to have disease. This approach may shorten the reporting time for patients requiring intervention, thereby improving clinical workflow efficiency. Nevertheless, further studies are warranted to refine diagnostic performance and to explore the feasibility of incorporating the model into structured reporting systems for routine clinical practice.

This study has several limitations. First, the reference standard used, which was based on consensus between two radiologists, may lack sufficient objectivity and consistency. While angiography was also considered in some cases to provide a more robust and standardized reference, it remains a two-dimensional imaging modality and its interpretation can still vary depending on the reader. Second, although temporal validation was performed through temporal separation, the study was conducted at a single center. As a result, its generalizability to other institutions or imaging protocols may be limited. Multicenter validation or testing across diverse imaging environments would help strengthen the clinical applicability of the model. Third, our study served as preliminary research, using a relatively coarse grading system (≥50% stenosis). More detailed grading may further enhance diagnostic capabilities. However, because the femoropopliteal artery is a small structure, detailed classification was highly subjective and lacked sufficient accuracy, making further subdivision difficult. Fourth, our retrospective study design introduces potential selection bias. Inherent data imbalance in clinical environments could have influenced model training and outcomes; however, we intentionally retained this distribution to reflect the true prevalence and class imbalance observed in real-world practice, thereby providing a more clinically relevant assessment of model performance. Finally, our study did not include a detailed calibration analysis. As proper model calibration is essential for clinical applicability, future work should incorporate calibration assessment to ensure that predicted probabilities accurately reflect the true risk.

In conclusion, our deep learning-based model demonstrated high accuracy in detecting significant steno-occlusion in peripheral arterial disease and acute limb ischemia patients-using computed tomography angiography maximum intensity projection images, and the integration of multi-angle analysis further improved diagnostic performance, highlighting the potential of artificial intelligence-assisted stenosis prediction in clinical practice.

## Figures and Tables

**Table 1:** Summary of patient demographics and stenosis distribution.

Dataset		Internal Dataset	Temporal Validation Dataset	*p*-Value
Number of cases		642	76	
Sex				0.699
- Male		472 (73.5%)	58 (76.3%)	
- Female		170 (26.5%)	18 (23.7%)	
Age				0.418
- Mean age ± Standard deviation		68.2 ± 13.5	70.1 ± 13.3	
- Range		16–98	20–96	
- Distribution				0.840
- ≤30 s		20 (3.1%)	2 (2.6%)	
- 40 s		40 (6.2%)	2 (2.6%)	
- 50 s		82 (12.8%)	9 (11.8%)	
- 60 s		180 (28.0%)	19 (25.0%)	
- 70 s		185 (28.8%)	25 (32.9%)	
- 80 s		119 (18.5%)	17 (22.4%)	
- 90 s		16 (2.5%)	2 (2.6%)	
Conventional angiography (within 1 month)		230 (35.82%)	22 (28.6%)	0.289
Significant steno-occlusion distribution by segment count				0.752
- No segment	(0/8)	336 (52.3%)	35 (46.1%)	
- At least one segment	(≥1/8)	306 (47.7%)	41 (53.9%)	
- One segment	(1/8)	55 (8.6%)	11 (14.5%)	
- Two segments	(2/8)	57 (8.9%)	5 (6.6%)	
- Three segments	(3/8)	48 (7.5%)	5 (6.6%)	
- Four segments	(4/8)	35 (5.5%)	4 (5.3%)	
- Five segments	(5/8)	33 (5.1%)	4 (5.3%)	
- Six segments	(6/8)	33 (5.1%)	4 (5.3%)	
- Seven segments	(7/8)	24 (3.7%)	5 (6.6%)	
- All eight segments	(8/8)	21 (3.3%)	3 (3.9%)	
Significant steno-occlusion distribution by location				0.897
- Rt. proximal SFA		129 (20.1%)	17 (22.4%)	
- Rt. mid SFA		156 (24.3%)	24 (31.6%)	
- Rt. distal SFA		169 (26.3%)	25 (32.9%)	
- Rt. PopA		124 (19.3%)	15 (19.7%)	
- Lt. proximal SFA		140 (21.8%)	16 (21.1%)	
- Lt. mid SFA		148 (23.1%)	25 (31.6%)	
- Lt. distal SFA		163 (25.4%)	18 (23.7%)	
- Lt. PopA		124 (19.3%)	16 (21.1%)	
Calcium degree				0.304
- No calcium		264 (41.1%)	26 (34.2%)	
- Low calcium		253 (39.4%)	30 (39.5%)	
- High calcium		125 (19.5%)	20 (26.3%)	

SFA = superficial femoral artery; PopA = popliteal artery.

**Table 2:** Performance of predicting significant steno-occlusion (SSO) in femoropopliteal artery using a single AP projection MIP.

Dataset	Deep Learning Model	Accuracy	Sensitivity	Specificity	PPV	NPV	F1-Score	AUC
Internal test set	DenseNet169	80.1% ± 3.8%	0.850 ± 0.070	0.701 ± 0.083	0.856 ± 0.028	0.705 ± 0.097	0.851 ± 0.032	0.874 ± 0.024
	EfficientNet-B6	74.9% ± 8.0%	0.754 ± 0.162	0.746 ± 0.152	0.865 ± 0.056	0.643 ± 0.161	0.793 ± 0.089	0.855 ± 0.048
	RDNet	82.6% ± 3.5%	0.873 ± 0.072	0.732 ± 0.078	0.872 ± 0.029	0.750 ± 0.114	0.870 ± 0.029	0.886 ± 0.031
Temporal validation dataset	DenseNet169	77.6%	0.900	0.639	0.735	0.852	0.809	0.881
	EfficientNet-B6	76.3%	0.750	0.778	0.789	0.737	0.769	0.835
	RDNet	77.6%	0.925	0.611	0.725	0.880	0.813	0.890

AP = anteroposterior; MIP = maximum intensity projection.

**Table 3:** Performance of predicting significant steno-occlusion based on the AUC and accuracy using multi-angle MIP images with four-segment rotational analysis.

Dataset	Approach	Deep Learning Model	Accuracy	Sensitivity	Specificity	PPV	NPV	F1-Score
Internal test set	Single	DenseNet169	87.8% ± 0.3%	0.898 ± 0.054	0.872 ± 0.018	0.687 ± 0.054	0.965 ± 0.021	0.777 ± 0.031
		EfficientNet-B6	85.5% ± 2.4%	0.879 ± 0.039	0.849 ± 0.043	0.648 ± 0.090	0.956 ± 0.017	0.742 ± 0.046
		RDNet	87.9% ± 2.1%	0.951 ± 0.052	0.857 ± 0.041	0.679 ± 0.071	0.981 ± 0.020	0.789 ± 0.028
	Half	DenseNet169	88.1% ± 2.8%	0.950 ± 0.028	0.864 ± 0.041	0.641 ± 0.089	0.986 ± 0.008	0.763 ± 0.058
		EfficientNet-B6	90.2% ± 2.4%	0.920 ± 0.031	0.897 ± 0.035	0.696 ± 0.077	0.979 ± 0.004	0.791 ± 0.051
		RDNet	89.3% ± 3.7%	0.952 ± 0.022	0.879 ± 0.048	0.669 ± 0.138	0.986 ± 0.008	0.780 ± 0.089
	Full	DenseNet169	87.9% ± 2.4%	0.948 ± 0.027	0.861 ± 0.035	0.636 ± 0.074	0.986 ± 0.005	0.760 ± 0.053
		EfficientNet-B6	90.0% ± 1.1%	0.923 ± 0.035	0.894 ± 0.015	0.686 ± 0.069	0.979 ± 0.006	0.786 ± 0.052
		RDNet	89.6% ± 1.5%	0.936 ± 0.025	0.887 ± 0.022	0.676 ± 0.087	0.982 ± 0.010	0.783 ± 0.054
Temporal validation dataset	Single	DenseNet169	84.7%	0.890	0.832	0.645	0.957	0.748
		EfficientNet-B6	87.3%	0.858	0.879	0.707	0.948	0.776
		RDNet	86.8%	0.897	0.859	0.685	0.960	0.777
	Half	DenseNet169	89.3%	0.865	0.903	0.753	0.951	0.805
		EfficientNet-B6	89.1%	0.832	0.912	0.763	0.941	0.796
		RDNet	89.0%	0.845	0.905	0.753	0.945	0.796
	Full	DenseNet169	88.7%	0.897	0.883	0.724	0.962	0.801
		EfficientNet-B6	89.5%	0.897	0.894	0.743	0.962	0.813
		RDNet	89.0%	0.877	0.894	0.739	0.955	0.802

**Table 4:** AUCs and comparison of AUCs of single-, half- and full-approaches for predicting significant steno-occlusion.

Dataset	Deep Learning Model	Approach			*p*-Value			
		Single	Half	Full	All *	Single-Half ^†^	Single-Full ^†^	Half-Full ^†^
Internal test set	DenseNet169	0.946 ± 0.013	0.961 ± 0.007	0.957 ± 0.009	0.002	0.005	0.049	0.25
	EfficientNet-B6	0.930 ± 0.009	0.959 ± 0.011	0.957 ± 0.014	<0.001	<0.001	<0.001	0.72
	RDNet	0.957 ± 0.015	0.964 ± 0.013	0.964 ± 0.009	0.30	0.25	0.25	1.00
Temporal validation dataset	DenseNet169	0.939	0.949	0.957	-	-	-	-
	EfficientNet-B6	0.925	0.940	0.949	-	-	-	-
	RDNet	0.940	0.959	0.954	-	-	-	-

* AUCs and accuracies of three different approaches were compared using ANOVA. ^†^ AUCs and accuracies of two approaches were compared using Mann-Whitney U test. AUC = area under the curve; MIP = maximum intensity projection.

**Table 5:** Predictive performance (accuracy) of the multi-region ensemble model according to the number of thresholds.

Dataset	Approach	Deep Learning Model	Threshold			
			Five	Six	Seven	Eight
Internal test set	Single	DenseNet169	95.4% ± 0.0%	89.2% ± 1.2%	74.8% ± 2.8%	54.1% ± 2.4%
		EfficientNet-B6	92.8% ± 0.8%	86.6% ± 2.0%	71.1% ± 2.5%	50.5% ± 1.0%
		RDNet	94.8% ± 2.0%	90.2% ± 2.0%	76.3% ± 0.7%	53.6% ± 0.3%
	Half	DenseNet169	95.9% ± 0.7%	90.7% ± 1.3%	76.3% ± 4.0%	61.3% ± 6.0%
		EfficientNet-B6	93.8% ± 2.2%	88.7% ± 3.1%	75.8% ± 7.2%	64.4% ± 7.6%
		RDNet	93.9% ± 2.4%	88.7% ± 1.8%	72.8% ± 5.5%	63.0% ± 3.9%
	Full	DenseNet169	92.3% ± 1.3%	85.5% ± 3.5%	72.6% ± 3.4%	57.2% ± 4.0%
		EfficientNet-B6	93.3% ± 0.7%	90.2% ± 1.6%	77.8% ± 2.9%	63.4% ± 3.1%
		RDNet	96.4% ± 0.7%	91.2% ± 0.6%	79.9% ± 2.3%	60.9% ± 1.9%
Temporal validation dataset	Single	DenseNet169	93.00%	88.20%	75.00%	53.10%
		EfficientNet-B6	92.10%	84.60%	73.20%	50.40%
		RDNet	93.90%	82.90%	69.30%	53.50%
	Half	DenseNet169	94.70%	90.80%	76.30%	56.10%
		EfficientNet-B6	93.40%	86.40%	71.90%	53.90%
		RDNet	93.40%	86.40%	71.90%	53.90%
	Full	DenseNet169	93.90%	88.20%	75.00%	57.90%
		EfficientNet-B6	95.60%	86.00%	73.20%	56.10%
		RDNet	95.60%	89.50%	75.40%	59.60%

**Table 6:** Subgroup analysis of prediction performance based on the AUC according to calcium grade.

Approach	No Calcium			Low Calcium			High Calcium			*p*-Value *
	DenseNet169	EfficientNet-B6	RDNet	DenseNet169	EfficientNet-B6	RDNet	DenseNet169	EfficientNet-B6	RDNet	
Single	0.958	0.935	0.982	0.961	0.949	0.965	0.834	0.787	0.829	<0.001
Half	0.962	0.972	0.985	0.969	0.964	0.963	0.823	0.842	0.870	<0.001
Full	0.987	0.979	0.968	0.962	0.953	0.968	0.836	0.858	0.841	<0.001

AUC = area under the curve. * AUCs of three different calcium grade groups were compared using ANOVA.

## Data Availability

The data presented in this study are available on request from the corresponding author due to ethical restrictions; access requires approval from the institutional ethics committee.

## Supplementary Materials

The following supporting information can be downloaded at: https://www.mdpi.com/article/10.3390/tomography11090104/s1. Table S1: Subgroup analysis of prediction performance based on the AUC according to age; Table S2: Subgroup analysis of prediction performance based on the AUC according to sex.
