# Supplementary material for: Deep Learning-Based Diagnosis of Femoropopliteal Artery Steno-Occlusion Using Maximum Intensity Projection Images of CT Angiography
Source: Tomography. 2025 Sep 8;11(9):104. doi: 10.3390/tomography11090104 (PMC12473302; doi:10.3390/tomography11090104)
Supplement: Supplementary file 1 [file tomography-11-00104-s001.zip › tomography-3808798-supplementary.pdf]

Supplementary table S1. Subgroup analysis of prediction performance based on the AUC according to age.

Dataset	Approach	≤50s			60s			70s			≥80s			*p*-value
		DenseNet169	EfficientNet-B6	RDNet	DenseNet169	EfficientNet-B6	RDNet	DenseNet169	EfficientNet-B6	RDNet	DenseNet169	EfficientNet-B6	RDNet	
Internal test set	Single	0.956 ± 0.000	0.891 ± 0.000	0.954 ± 0.000	0.956 ± 0.025	0.947 ± 0.034	0.925 ± 0.057	0.908 ± 0.113	0.904 ± 0.053	0.906 ± 0.097	0.941 ± 0.046	0.921 ± 0.059	0.978 ± 0.017	0.018
	Half	0.941 ± 0.019	0.953 ± 0.020	0.952 ± 0.029	0.991 ± 0.009	0.975 ± 0.023	0.959 ± 0.024	0.922 ± 0.034	0.965 ± 0.010	0.948 ± 0.023	0.980 ± 0.012	0.971 ± 0.018	0.989 ± 0.004	<0.001
	Full	0.921 ± 0.062	0.936 ± 0.013	0.949 ± 0.029	0.951 ± 0.027	0.979 ± 0.017	0.980 ± 0.028	0.968 ± 0.007	0.951 ± 0.012	0.954 ± 0.004	0.971 ± 0.011	0.987 ± 0.002	0.978 ± 0.023	0.027
Temporal validation dataset	Single	0.881	0.815	0.882	0.982	0.965	0.977	0.929	0.924	0.943	0.973	0.953	0.967	0.042
	Half	0.908	0.874	0.946	0.981	0.971	0.980	0.935	0.941	0.957	0.975	0.974	0.980	0.011
	Full	0.915	0.904	0.928	0.98	0.976	0.975	0.946	0.935	0.941	0.983	0.983	0.973	<0.011

Supplementary table S2. Subgroup analysis of prediction performance based on the AUC according to sex.

Dataset	Approach	Male			Female			*p*-value
		DenseNet169	EfficientNet-B6	RDNet	DenseNet169	EfficientNet-B6	RDNet	
Internal test set	Single	0.946 ± 0.015	0.926 ± 0.011	0.954 ± 0.014	0.962 ± 0.013	0.968 ± 0.008	0.982 ± 0.002	0.032
	Half	0.958 ± 0.006	0.958 ± 0.009	0.965 ± 0.013	0.990 ± 0.006	0.982 ± 0.019	0.987 ± 0.007	0.021
	Full	0.955 ± 0.009	0.955 ± 0.013	0.965 ± 0.010	0.980 ± 0.021	0.979 ± 0.018	0.980 ± 0.017	0.045
Temporal validation dataset	Single	0.949	0.93	0.951	0.931	0.941	0.958	<0.001
	Half	0.955	0.946	0.966	0.951	0.948	0.965	0.012
	Full	0.965	0.954	0.957	0.952	0.951	0.960	0.018
